# Bis(1,10-phenanthroline-κ^2^ *N*,*N*′)(sulfato-κ^2^ *O*,*O*′)nickel(II) propane-1,2-diol monosolvate

**DOI:** 10.1107/S1600536813023027

**Published:** 2013-08-21

**Authors:** Kai-Long Zhong, Cheng-Xian Duan

**Affiliations:** a Department of Applied Chemistry, Nanjing College of Chemical Technology, Nanjing, 210048, People’s Republic of China; b AVIC Hefei Jianghang Aircraft Equipment Corporation Ltd, Hefei, 230051, People’s Republic of China

## Abstract

In the title compound, [Ni(SO_4_)(C_12_H_8_N_2_)_2_]·C_3_H_8_O_2_, the Ni^II^ atom exhibits a distorted octa­hedral coordination by four N atoms from two chelating 1,10-phenanthroline ligands and two O atoms from an *O*,*O*′-bidentate sulfate group. A twofold rotation axis passes through the Ni and S atoms and the mid-point of the hydroxyl C—C bond of the propane-1,2-diol solvent mol­ecule. The dihedral angle between the two chelating N_2_C_2_ groups is 85.61 (8)°. The [NiSO_4_(C_10_H_8_N_2_)_2_] and propane-1,2-diol units are held together by a pair of symmetry-related inter­molecular O—H⋯O hydrogen bonds involving the uncoordinating O atoms of the sulfate ion. Due to symmetry, the solvent mol­ecule is equally disordered over two positions.

## Related literature

For the ethane-1,2-diol solvate of the title complex, see: Zhong *et al.* (2009 ▶). For the propane-1,3-diol solvate of the title complex, see: Ni *et al.* (2010 ▶). For the butane-2,3-diol solvate of the title complex, see: Zhong & Ni (2012 ▶). For an isotypic compound, see: Zhong (2013 ▶). For background to coordination polymers, see: Batten & Robson (1998 ▶); Zhang *et al.* (2010 ▶); Zhong *et al.* (2011 ▶).
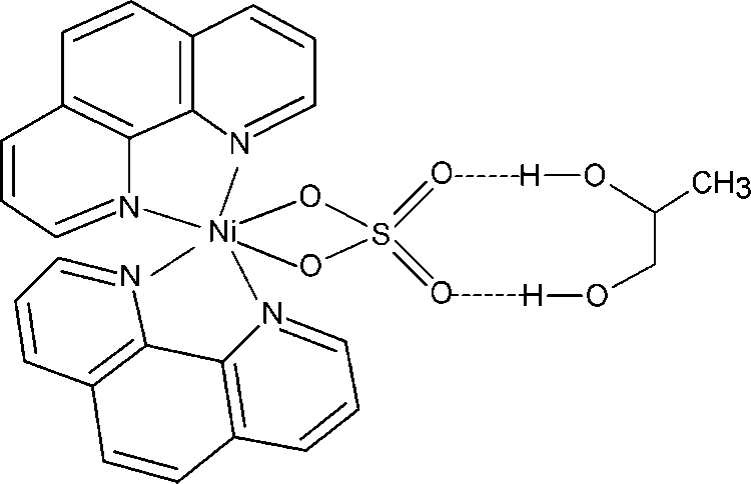


## Experimental

### 

#### Crystal data

[Ni(SO_4_)(C_12_H_8_N_2_)_2_]·C_3_H_8_O_2_
*M* *_r_* = 591.27
Monoclinic, 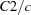

*a* = 18.0277 (10) Å
*b* = 13.0448 (5) Å
*c* = 12.8070 (5) Å
β = 121.738 (5)°
*V* = 2561.4 (2) Å^3^
*Z* = 4
Mo *K*α radiation
μ = 0.89 mm^−1^
*T* = 223 K
0.30 × 0.25 × 0.15 mm

#### Data collection

Rigaku Mercury CCD diffractometer
Absorption correction: multi-scan (*REQAB*; Jacobson, 1998 ▶) *T*_min_ = 0.876, *T*_max_ = 1.000
7993 measured reflections
2602 independent reflections
2311 reflections with *I* > 2σ(*I*)
*R*_int_ = 0.031

#### Refinement

*R*[*F* ^2^ > 2σ(*F* ^2^)] = 0.034
*wR*(*F* ^2^) = 0.084
*S* = 1.01
2602 reflections
191 parameters
26 restraints
H-atom parameters constrained
Δρ_max_ = 0.32 e Å^−3^
Δρ_min_ = −0.42 e Å^−3^

### 

Data collection: *CrystalClear* (Rigaku, 2007 ▶); cell refinement: *CrystalClear*; data reduction: *CrystalClear*; program(s) used to solve structure: *SHELXS97* (Sheldrick, 2008 ▶); program(s) used to refine structure: *SHELXL97* (Sheldrick, 2008 ▶); molecular graphics: *XP* in *SHELXTL* (Sheldrick, 2008 ▶); software used to prepare material for publication: *SHELXTL*.

## Supplementary Material

Crystal structure: contains datablock(s) global, I. DOI: 10.1107/S1600536813023027/zq2207sup1.cif

Structure factors: contains datablock(s) I. DOI: 10.1107/S1600536813023027/zq2207Isup2.hkl

Additional supplementary materials:  crystallographic information; 3D view; checkCIF report

## Figures and Tables

**Table 1:** Selected bond lengths (Å)

Ni1—N1	2.0762 (19)
Ni1—N2	2.082 (2)
Ni1—O1	2.1074 (16)
S1—O2	1.4587 (17)
S1—O1	1.4942 (16)

**Table 2:** Hydrogen-bond geometry (Å, °)

*D*—H⋯*A*	*D*—H	H⋯*A*	*D*⋯*A*	*D*—H⋯*A*
O3—H3*B*⋯O2	0.82	1.97	2.705 (6)	148
O3′—H3′*A*⋯O2	0.82	2.00	2.767 (8)	155

**Fig. 1.**
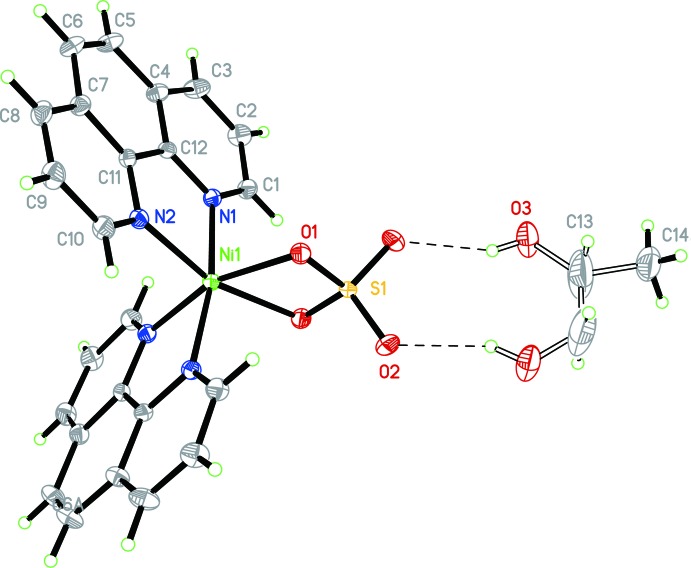
The molecular structure showing the atom-numbering scheme and with displacement ellipsoids drawn at the 30% probability level. The light broken lines depict O—H···O interactions. Unlabeled atoms are related to the labelled atoms by the symmetry operator (-x, y, -z + 1/2).

## supplementary crystallographic information

### 1. Comment

The self-assembly of coordination polymers and the crystal engineering of metal-organic coordination frameworks have recently attracted great interest, owing to their interesting structural topologies and potential application as functional materials (Batten & Robson, 1998; Zhang *et al.*, 2010; Zhong *et al.*, 2011). The neutral bidentate ligand 1,10-phenanthroline (phen) as an auxiliary ligand has been widely applied in constructing interesting coordination polymers. Recently, we have obtained unexpectedly some nickel-phen complexes (Zhong *et al.*, 2009; Ni *et al.*, 2010; Zhong & Ni, 2012) with interesting four-membered chelating rings during attempts to synthesize mixed-ligand coordination polymers with phen as auxiliary ligand *via* an alcohol-solvothermal reaction. We here report the title compound, [NiSO_4_(C_12_H_8_N_2_)_2_]. C_3_H_8_O_2_, which is the part of our systematic investigation of transition metal nickel complexes with bidentate bridging sulfate ligands. It is isostructural to the previously reported cobalt(II) analog (Zhong, 2013).

The single-crystal X-ray diffraction experiment revealed that the crystal structure of the the title compound consists of a neutral monomeric [NiSO_4_(C_10_H_8_N_2_)_2_] complex and a solvent propane-1,2-diol molecule. A two-fold rotation axis (symmetry code: -*x* + 1, *y*, -*z* + 1/2) passes through the Ni and S atoms, and the mid-point of the hydroxyl C—C bond of the propane-1,2-diol solvent molecule is likewise located on a the same crystallographic axis. The Ni^II^ metal ion has a distorted NiN_4_O_2_ octahedral geometry, with four N atoms from two chelating phenanthroline ligands and two O atoms from an *O*,*O*'-bidentate sulfate anion (Fig. 1). The Ni—O bond distance of 2.107 (2) Å, the O—Ni—O bite angle of 67.95 (9)°, the Ni—N bond distances in the range of 2.076 (2)–2.082 (2) Å, the N—Ni—N bite angle of 80.09 (7)° and the dihedral angle of 85.61 (8)° between the two chelating NCCN groups are in good agreement with those observed in the previously reported nickel complexes (Zhong *et al.*, 2009; Ni *et al.*, 2010; Zhong & Ni, 2012) (Table 1).

The solvent molecule is disordered over two positions and was refined with a site-occupancy ratio of 0.50:0.50. The metal complex and the solvent molecules are held together by a pair of intermolecular O—H···O hydrogen bonds, which help to further stabilize the crystal structure (Fig.1 and Table 2).

### 2. Experimental

Green block-shaped crystals of the title compound were obtained by a procedure similar to that described previously (Zhong, 2013), but with NiSO_4_·7H_2_O in place of CoSO_4_·7H_2_O.

### 3. Refinement

The non-hydrogen atoms were refined anisotropically. The H atoms of phen were positioned geometrically and allowed to ride on their parent atoms, with C—H = 0.93 Å and *U*_iso_(H) = 1.2*U*_eq_(C). The H atoms of propane-1,2-diol were placed in geometrically idealized positions and refined as riding atoms, with C—H(CH_3_) = 0.96 Å, C—H(CH_2_) = 0.97 Å, C—H(CH) = 0.98 Å and O—H = 0.82 Å; *U*_iso_(H) = 1.2*U*_eq_(C) and 1.5*U*_eq_(O). The solvent molecule butane-2,3-diol is disordered over two positions and was refined with 0.50 and 0.50 site occupancies.

### Crystal data

**Table:**

[Ni(SO_4_)(C_12_H_8_N_2_)_2_]·C_3_H_8_O_2_	*F*(000) = 1224
*M**_r_* = 591.27	*D*_x_ = 1.533 Mg m^−^^3^
Monoclinic, *C*2/*c*	Mo *K*α radiation, λ = 0.71073 Å
Hall symbol: -C 2yc	Cell parameters from 3439 reflections
*a* = 18.0277 (10) Å	θ = 3.6–28.8°
*b* = 13.0448 (5) Å	µ = 0.89 mm^−^^1^
*c* = 12.8070 (5) Å	*T* = 223 K
β = 121.738 (5)°	Block, green
*V* = 2561.4 (2) Å^3^	0.30 × 0.25 × 0.15 mm
*Z* = 4	

### Data collection

**Table:**

Rigaku Mercury CCD diffractometer	2602 independent reflections
Radiation source: fine-focus sealed tube	2311 reflections with *I* > 2σ(*I*)
Graphite Monochromator monochromator	*R*_int_ = 0.031
Detector resolution: 28.5714 pixels mm^-1^	θ_max_ = 26.4°, θ_min_ = 3.1°
ω scans	*h* = −21→22
Absorption correction: multi-scan (*REQAB*; Jacobson, 1998)	*k* = −16→16
*T*_min_ = 0.876, *T*_max_ = 1.000	*l* = −16→14
7993 measured reflections	

### Refinement

**Table:**

Refinement on *F*^2^	Primary atom site location: structure-invariant direct methods
Least-squares matrix: full	Secondary atom site location: difference Fourier map
*R*[*F*^2^ > 2σ(*F*^2^)] = 0.034	Hydrogen site location: inferred from neighbouring sites
*wR*(*F*^2^) = 0.084	H-atom parameters constrained
*S* = 1.01	*w* = 1/[σ^2^(*F*_o_^2^) + (0.0343*P*)^2^ + 4.7899*P*] where *P* = (*F*_o_^2^ + 2*F*_c_^2^)/3
2602 reflections	(Δ/σ)_max_ < 0.001
191 parameters	Δρ_max_ = 0.32 e Å^−^^3^
26 restraints	Δρ_min_ = −0.42 e Å^−^^3^

### Special details

**Table:**

Geometry. All e.s.d.'s (except the e.s.d. in the dihedral angle between two l.s. planes) are estimated using the full covariance matrix. The cell e.s.d.'s are taken into account individually in the estimation of e.s.d.'s in distances, angles and torsion angles; correlations between e.s.d.'s in cell parameters are only used when they are defined by crystal symmetry. An approximate (isotropic) treatment of cell e.s.d.'s is used for estimating e.s.d.'s involving l.s. planes.
Refinement. Refinement of *F*^2^ against ALL reflections. The weighted *R*-factor *wR* and goodness of fit *S* are based on *F*^2^, conventional *R*-factors *R* are based on *F*, with *F* set to zero for negative *F*^2^. The threshold expression of *F*^2^ > σ(*F*^2^) is used only for calculating *R*-factors(gt) *etc*. and is not relevant to the choice of reflections for refinement. *R*-factors based on *F*^2^ are statistically about twice as large as those based on *F*, and *R*- factors based on ALL data will be even larger.

### Fractional atomic coordinates and isotropic or equivalent isotropic displacement parameters (Å^2^)

**Table:**

	*x*	*y*	*z*	*U*_iso_*/*U*_eq_	Occ. (<1)
Ni1	0.0000	0.18120 (3)	0.2500	0.02136 (14)	
S1	0.0000	−0.02326 (6)	0.2500	0.02110 (19)	
O1	0.05906 (10)	0.04723 (12)	0.35253 (14)	0.0273 (4)	
O2	−0.05048 (12)	−0.08688 (14)	0.28419 (17)	0.0364 (4)	
O3	−0.0543 (5)	−0.2918 (4)	0.3137 (6)	0.0602 (19)	0.50
H3B	−0.0440	−0.2379	0.2907	0.090*	0.50
O3'	−0.0830 (5)	−0.2955 (5)	0.2536 (7)	0.083 (3)	0.50
H3'A	−0.0580	−0.2399	0.2694	0.124*	0.50
N1	0.08057 (13)	0.19506 (14)	0.18013 (17)	0.0236 (4)	
N2	0.09201 (13)	0.28362 (15)	0.37649 (18)	0.0245 (4)	
C1	0.07474 (16)	0.14850 (19)	0.0836 (2)	0.0288 (5)	
H1A	0.0310	0.1001	0.0411	0.035*	
C2	0.13188 (18)	0.1696 (2)	0.0438 (2)	0.0358 (6)	
H2A	0.1258	0.1360	−0.0244	0.043*	
C3	0.19674 (17)	0.2399 (2)	0.1056 (2)	0.0373 (6)	
H3A	0.2352	0.2542	0.0799	0.045*	
C4	0.20528 (16)	0.2908 (2)	0.2082 (2)	0.0285 (5)	
C5	0.27283 (17)	0.3631 (2)	0.2805 (2)	0.0361 (6)	
H5A	0.3117	0.3819	0.2569	0.043*	
C6	0.28081 (17)	0.4044 (2)	0.3826 (2)	0.0340 (6)	
H6A	0.3259	0.4503	0.4291	0.041*	
C7	0.22142 (16)	0.37889 (19)	0.4206 (2)	0.0277 (5)	
C8	0.22877 (17)	0.41661 (19)	0.5286 (2)	0.0331 (6)	
H8A	0.2738	0.4612	0.5796	0.040*	
C9	0.16923 (19)	0.3871 (2)	0.5578 (2)	0.0363 (6)	
H9A	0.1736	0.4110	0.6293	0.044*	
C10	0.10143 (18)	0.32078 (19)	0.4793 (2)	0.0318 (6)	
H10A	0.0611	0.3019	0.5003	0.038*	
C11	0.15246 (15)	0.31125 (16)	0.3486 (2)	0.0218 (5)	
C12	0.14502 (14)	0.26499 (17)	0.2415 (2)	0.0223 (5)	
C13	−0.0251 (5)	−0.3713 (3)	0.2775 (7)	0.133 (3)	0.50
H13	−0.0796	−0.3735	0.1972	0.160*	0.50
C13'	−0.0251 (5)	−0.3713 (3)	0.2775 (7)	0.133 (3)	0.50
H13A	−0.0571	−0.4354	0.2556	0.160*	0.50
H13B	0.0153	−0.3727	0.3657	0.160*	0.50
C14	−0.0365 (6)	−0.4760 (5)	0.3032 (10)	0.088 (3)	0.50
H14A	−0.0732	−0.4774	0.3366	0.133*	0.50
H14B	0.0193	−0.5050	0.3613	0.133*	0.50
H14C	−0.0631	−0.5151	0.2286	0.133*	0.50

### Atomic displacement parameters (Å^2^)

**Table:**

	*U*^11^	*U*^22^	*U*^33^	*U*^12^	*U*^13^	*U*^23^
Ni1	0.0205 (2)	0.0205 (2)	0.0254 (2)	0.000	0.01365 (18)	0.000
S1	0.0167 (4)	0.0205 (4)	0.0254 (4)	0.000	0.0106 (3)	0.000
O1	0.0222 (8)	0.0259 (9)	0.0246 (8)	0.0005 (7)	0.0059 (7)	−0.0006 (7)
O2	0.0335 (10)	0.0345 (10)	0.0475 (11)	−0.0061 (8)	0.0258 (9)	0.0048 (8)
O3	0.107 (5)	0.034 (3)	0.082 (4)	−0.010 (3)	0.079 (4)	−0.009 (3)
O3'	0.106 (5)	0.054 (4)	0.120 (6)	−0.040 (4)	0.082 (5)	−0.040 (4)
N1	0.0234 (10)	0.0231 (10)	0.0254 (10)	−0.0011 (8)	0.0137 (9)	−0.0028 (8)
N2	0.0265 (10)	0.0228 (10)	0.0283 (10)	0.0005 (8)	0.0172 (9)	−0.0009 (8)
C1	0.0290 (13)	0.0301 (13)	0.0267 (12)	−0.0032 (11)	0.0143 (11)	−0.0062 (10)
C2	0.0384 (15)	0.0460 (16)	0.0288 (13)	−0.0028 (13)	0.0216 (12)	−0.0084 (12)
C3	0.0333 (14)	0.0554 (18)	0.0323 (13)	−0.0071 (13)	0.0235 (12)	−0.0029 (13)
C4	0.0251 (12)	0.0357 (13)	0.0259 (12)	−0.0036 (11)	0.0142 (10)	0.0011 (10)
C5	0.0275 (13)	0.0482 (16)	0.0349 (14)	−0.0115 (12)	0.0182 (12)	0.0007 (12)
C6	0.0260 (13)	0.0388 (15)	0.0318 (13)	−0.0122 (11)	0.0114 (11)	−0.0031 (11)
C7	0.0260 (12)	0.0265 (12)	0.0265 (11)	−0.0014 (10)	0.0109 (10)	0.0010 (10)
C8	0.0334 (14)	0.0293 (13)	0.0295 (13)	−0.0051 (11)	0.0117 (11)	−0.0068 (11)
C9	0.0463 (16)	0.0357 (14)	0.0310 (13)	−0.0027 (13)	0.0230 (13)	−0.0101 (11)
C10	0.0374 (14)	0.0315 (13)	0.0338 (13)	−0.0017 (11)	0.0239 (12)	−0.0050 (11)
C11	0.0218 (11)	0.0196 (11)	0.0236 (11)	0.0008 (9)	0.0116 (10)	0.0017 (9)
C12	0.0197 (11)	0.0228 (11)	0.0240 (11)	0.0011 (9)	0.0112 (9)	0.0020 (9)
C13	0.227 (7)	0.043 (2)	0.242 (7)	−0.020 (4)	0.201 (7)	−0.011 (4)
C13'	0.227 (7)	0.043 (2)	0.242 (7)	−0.020 (4)	0.201 (7)	−0.011 (4)
C14	0.107 (7)	0.040 (4)	0.155 (9)	0.000 (4)	0.093 (7)	0.001 (5)

### Geometric parameters (Å, º)

**Table:**

Ni1—N1	2.0762 (19)	C3—H3A	0.9300
Ni1—N1^i^	2.0762 (19)	C4—C12	1.401 (3)
Ni1—N2	2.082 (2)	C4—C5	1.430 (4)
Ni1—N2^i^	2.082 (2)	C5—C6	1.350 (4)
Ni1—O1^i^	2.1074 (16)	C5—H5A	0.9300
Ni1—O1	2.1074 (16)	C6—C7	1.429 (3)
S1—O2^i^	1.4587 (17)	C6—H6A	0.9300
S1—O2	1.4587 (17)	C7—C11	1.403 (3)
S1—O1	1.4942 (16)	C7—C8	1.408 (3)
S1—O1^i^	1.4942 (16)	C8—C9	1.364 (4)
O3—C13	1.350 (4)	C8—H8A	0.9300
O3—H3B	0.8200	C9—C10	1.400 (4)
O3'—H3'A	0.8200	C9—H9A	0.9300
N1—C1	1.331 (3)	C10—H10A	0.9300
N1—C12	1.356 (3)	C11—C12	1.439 (3)
N2—C10	1.328 (3)	C13—C13^i^	1.411 (6)
N2—C11	1.362 (3)	C13—C14	1.444 (7)
C1—C2	1.397 (4)	C13—H13	0.9800
C1—H1A	0.9300	C14—H14A	0.9600
C2—C3	1.365 (4)	C14—H14B	0.9600
C2—H2A	0.9300	C14—H14C	0.9600
C3—C4	1.406 (3)		
			
N1—Ni1—N1^i^	170.01 (11)	C12—C4—C5	119.6 (2)
N1—Ni1—N2	80.09 (7)	C3—C4—C5	123.6 (2)
N1^i^—Ni1—N2	93.46 (7)	C6—C5—C4	120.7 (2)
N1—Ni1—N2^i^	93.46 (7)	C6—C5—H5A	119.6
N1^i^—Ni1—N2^i^	80.09 (7)	C4—C5—H5A	119.6
N2—Ni1—N2^i^	100.15 (11)	C5—C6—C7	121.3 (2)
N1—Ni1—O1^i^	92.43 (7)	C5—C6—H6A	119.3
N1^i^—Ni1—O1^i^	95.86 (7)	C7—C6—H6A	119.3
N2—Ni1—O1^i^	162.12 (7)	C11—C7—C8	117.3 (2)
N2^i^—Ni1—O1^i^	96.48 (7)	C11—C7—C6	119.1 (2)
N1—Ni1—O1	95.86 (7)	C8—C7—C6	123.6 (2)
N1^i^—Ni1—O1	92.43 (7)	C9—C8—C7	119.3 (2)
N2—Ni1—O1	96.48 (7)	C9—C8—H8A	120.3
N2^i^—Ni1—O1	162.12 (7)	C7—C8—H8A	120.3
O1^i^—Ni1—O1	67.95 (9)	C8—C9—C10	119.6 (2)
O2^i^—S1—O2	110.65 (16)	C8—C9—H9A	120.2
O2^i^—S1—O1	110.20 (10)	C10—C9—H9A	120.2
O2—S1—O1	110.79 (10)	N2—C10—C9	123.0 (2)
O2^i^—S1—O1^i^	110.79 (10)	N2—C10—H10A	118.5
O2—S1—O1^i^	110.20 (10)	C9—C10—H10A	118.5
O1—S1—O1^i^	104.03 (13)	N2—C11—C7	123.3 (2)
S1—O1—Ni1	94.01 (8)	N2—C11—C12	116.9 (2)
C13—O3—H3B	109.5	C7—C11—C12	119.7 (2)
C1—N1—C12	118.1 (2)	N1—C12—C4	123.4 (2)
C1—N1—Ni1	128.99 (17)	N1—C12—C11	117.2 (2)
C12—N1—Ni1	112.89 (14)	C4—C12—C11	119.4 (2)
C10—N2—C11	117.4 (2)	O3—C13—C13^i^	129.8 (4)
C10—N2—Ni1	129.79 (17)	O3—C13—C14	121.4 (5)
C11—N2—Ni1	112.70 (14)	C13^i^—C13—C14	108.6 (4)
N1—C1—C2	122.5 (2)	O3—C13—H13	91.5
N1—C1—H1A	118.8	C13^i^—C13—H13	91.5
C2—C1—H1A	118.8	C14—C13—H13	91.5
C3—C2—C1	119.5 (2)	C13—C14—H14A	109.5
C3—C2—H2A	120.3	C13—C14—H14B	109.5
C1—C2—H2A	120.3	H14A—C14—H14B	109.5
C2—C3—C4	119.9 (2)	C13—C14—H14C	109.5
C2—C3—H3A	120.0	H14A—C14—H14C	109.5
C4—C3—H3A	120.0	H14B—C14—H14C	109.5
C12—C4—C3	116.7 (2)		
			
O2^i^—S1—O1—Ni1	118.81 (10)	C12—C4—C5—C6	2.1 (4)
O2—S1—O1—Ni1	−118.40 (9)	C3—C4—C5—C6	−175.7 (3)
O1^i^—S1—O1—Ni1	0.0	C4—C5—C6—C7	−1.2 (4)
N1—Ni1—O1—S1	−90.25 (8)	C5—C6—C7—C11	−1.4 (4)
N1^i^—Ni1—O1—S1	95.34 (8)	C5—C6—C7—C8	177.5 (3)
N2—Ni1—O1—S1	−170.90 (8)	C11—C7—C8—C9	−0.6 (4)
N2^i^—Ni1—O1—S1	30.8 (3)	C6—C7—C8—C9	−179.4 (2)
O1^i^—Ni1—O1—S1	0.0	C7—C8—C9—C10	−0.6 (4)
N2—Ni1—N1—C1	178.4 (2)	C11—N2—C10—C9	0.9 (4)
N2^i^—Ni1—N1—C1	−81.9 (2)	Ni1—N2—C10—C9	177.70 (19)
O1^i^—Ni1—N1—C1	14.7 (2)	C8—C9—C10—N2	0.5 (4)
O1—Ni1—N1—C1	82.8 (2)	C10—N2—C11—C7	−2.2 (3)
N2—Ni1—N1—C12	−4.41 (15)	Ni1—N2—C11—C7	−179.53 (18)
N2^i^—Ni1—N1—C12	95.30 (16)	C10—N2—C11—C12	175.8 (2)
O1^i^—Ni1—N1—C12	−168.06 (15)	Ni1—N2—C11—C12	−1.5 (2)
O1—Ni1—N1—C12	−99.98 (15)	C8—C7—C11—N2	2.1 (3)
N1—Ni1—N2—C10	−173.8 (2)	C6—C7—C11—N2	−179.0 (2)
N1^i^—Ni1—N2—C10	13.9 (2)	C8—C7—C11—C12	−175.9 (2)
N2^i^—Ni1—N2—C10	94.4 (2)	C6—C7—C11—C12	3.0 (3)
O1^i^—Ni1—N2—C10	−107.5 (3)	C1—N1—C12—C4	0.3 (3)
O1—Ni1—N2—C10	−78.9 (2)	Ni1—N1—C12—C4	−177.28 (18)
N1—Ni1—N2—C11	3.16 (15)	C1—N1—C12—C11	−177.4 (2)
N1^i^—Ni1—N2—C11	−169.16 (16)	Ni1—N1—C12—C11	5.0 (2)
N2^i^—Ni1—N2—C11	−88.62 (15)	C3—C4—C12—N1	−0.2 (4)
O1^i^—Ni1—N2—C11	69.5 (3)	C5—C4—C12—N1	−178.1 (2)
O1—Ni1—N2—C11	98.00 (15)	C3—C4—C12—C11	177.5 (2)
C12—N1—C1—C2	−0.4 (4)	C5—C4—C12—C11	−0.4 (4)
Ni1—N1—C1—C2	176.71 (19)	N2—C11—C12—N1	−2.4 (3)
N1—C1—C2—C3	0.4 (4)	C7—C11—C12—N1	175.7 (2)
C1—C2—C3—C4	−0.3 (4)	N2—C11—C12—C4	179.8 (2)
C2—C3—C4—C12	0.2 (4)	C7—C11—C12—C4	−2.1 (3)
C2—C3—C4—C5	178.0 (3)		

Symmetry code: (i) −*x*, *y*, −*z*+1/2.

### Hydrogen-bond geometry (Å, º)

**Table:**

*D*—H···*A*	*D*—H	H···*A*	*D*···*A*	*D*—H···*A*
O3—H3*B*···O2	0.82	1.97	2.705 (6)	148
O3′—H3′*A*···O2	0.82	2.00	2.767 (8)	155
